# Long-term care (LTC) policy in Thailand on the homebound and bedridden elderly happiness

**DOI:** 10.1016/j.hpopen.2020.100026

**Published:** 2021-01-08

**Authors:** Savinee Suriyanrattakorn, Chia-Lin Chang

**Affiliations:** a Department of Applied Economics, National Chung Hsing University, Taiwan; b Department of Finance, National Chung Hsing University, Taiwan

**Keywords:** Long-term care, Homebound and bedridden elderly, Life satisfaction, Happiness

## Abstract

The number of homebound and bedridden elderly has been increasing in Thailand, as the aging population rapidly grows and rates of chronic diseases increase. However, decreasing family size may reflect a decline in the ability of families to provide care. Society, as a result, enhances the need for long-term care (LTC) policy to provide home care and social support for the homebound and bedridden elderly. This paper examines how care-receipt satisfaction in LTC impacts the homebound and bedridden elderly's overall happiness, using a two-year panel of 279 individuals from the Thai Health Promotion Foundation dataset. We use the pooled two-stage least square (Pooled-2SLS) model and random effect two-stage least square (RE-2SLS) model to control the endogeneity problem. The empirical results show that care-receipt satisfaction on LTC service can generate a positive impact on the overall happiness of the homebound and bedridden elderly.

## Highlights

• Examine LTC impact on the homebound and bedridden elderly’s life satisfaction.
• This paper mainly focused on tracking changes in subjective well-being outcome.
• Assessing the effectiveness of the long term care service on life satisfaction.
• Control the endogeneity issues of the care-receipt satisfaction.
• LTC play the most contribution to the well-being of bedridden elderly.

## 1 Introduction

While Thailand's total population was slowly increasing at approximately 0.21% in 2018, the elderly (aged 60 or over) population grew at a very high rate of around 4%. Compared to other Asian countries, Thailand ranks second after Singapore, with the highest proportion of aging at about 11 million people or 16.5 percent of the total population. The increase of the aging population and age-related chronic diseases also causes a rise in the number of homebound and bedridden elderly. The National Health Security office showed that there are approximately a million people who are so-called homebound and bedridden, and the number is increasing.

The Elderly can be categorized based on their functional and physical capacity using the Barthel Index of Activities of Daily Living (ADLs). These groups are as follows: active (ADLs more than 11), homebound (ADLs 5–11), and bedridden elderly (ADLs 0–4). Each group has particular care needs. For example, a healthier elder may not require any assistance to perform daily activities, while the bedridden and homebound may need more support in their daily routines. Homebound and bedridden elderly are the focus of this study because they are more prone to a variety of chronic diseases or disabilities than the active ones.

Moreover, as economic and demographic changes occur, family size is getting smaller, and approximately one-third of the elderly in Thailand have incomes lower than the poverty line [1]. In other words, an increase in the number of bedridden and homebound elderly are facing financial vulnerability at the same time the number of family caregivers is declining. This has resulted in Thailand's expansion of the need for long-term care policy to establish a proper and effective system to support the dependent elderly.

Recently, national long-term care policies for the elderly have received more attention in many countries, especially fast-aging countries like Thailand. Under the second “National Plan on the Elderly (2002–2021)”, the Thai government has put considerably more effort into establishing and developing a long-term care scheme to support the elderly with disabilities. In the year 2016, the government provided a proactive action in developing long-term care (LTC) system and identifies steps forward from regular nursing home care to community-based long-term care. The community-based system is provided through institutions and in communities, which combine cooperation among elderly's families, hospitals, volunteers for elderly, elderly clubs, village health volunteers, temples, schools, and local administrative offices. The development of LTC scheme for dependent elderly since the year 2010 to the proactive one in 2016 aims to ensure that the homebound and bedridden elderly will be satisfied with the appropriate primary care, social support, and, therefore happier in their life.

This study attempts to investigate whether higher care-receipt satisfaction in LTC causes greater overall happiness of dependent elderly. The key independent variable in this study is care-receipt satisfaction in LTC, which can be used as the proxy to represent LTC service quality. [2] Prakash mentions that patient satisfaction is a vital indicator for measuring healthcare quality. The link between satisfaction in health services and happiness is varied depending on the type of health services [3], [4], [5] state that happiness or satisfaction of life is one of the most aspiring LTC policy goals. Also, [6] suggests that subjective wellbeing should be one of the primary outcomes of appraising healthcare policy. Most previous research related to the impact of LTC on the elderly's physical functioning shows a positive relationship [7], [8]. However, only a few studies analyze the impact of healthcare care satisfaction on elderly subjective wellbeing [3], [4], [7].

This paper differs from other existing studies on LTC with two points: (1) this paper primarily focuses on tracking changes in subjective wellbeing, (2) this study aims to fill research gaps by analyzing a cause-and-effect relationship between LTC satisfaction and overall happiness through the use of a panel dataset representing a developing country. The rest of the paper is organized as follows: Section 2 mentions the LTC system for the elderly in Thailand, Section 3 presents the data and key variable descriptions, Section 4 performs empirical methods, and the final two sections show the empirical result, conclusions, and discussion.

## 2 Long term care system for the homebound and bedridden elderly in Thailand

According to the definition of long-term care defined by the National Institute on Aging (NIH) “the services that help people live as independently and as safely as possible when they can no longer perform everyday activities on their own”, LTC may perform whether by formal (often specialized and paid) or informal (often unspecialized and unpaid) care providers. In Thailand, most long-term care is provided informally by unpaid caregivers, such as family members or relatives. In the past, health care and social support systems for seniors with disabilities provided a limited amount of funds and services, and most long-term care facilities were public foster homes [8].

The concept of a national long-term care program for the elderly first appeared in 2010, as can be seen in the resolution of the second National Health Assembly. Afterward, in 2016, Thailand introduced a proactive long-term care scheme based on the concept of community-based long-term care (CLTC). Since then, the government started a trial proactive LTC program with a budget of 600 million Baht (covering 1000 sub-districts in 2016) and increased the budget to 900 and 1159 million Baht for the two following years. This additional funding was allocated to the National Health Security Office (NHSO) and included not only health care for the elderly but multiple care settings (such as nursing services, home visits, community-based volunteers, and providing some medical auxiliaries/equipment). The proactive plan emphasizes the development of a data system for registration, personal care planning, and reimbursement, as well as providing caregivers with adequate knowledge to deliver proper care to dependent elderly. Our target area of the study, “Udonthani province,” was selected as one of the pilot sites before applying proactive LTC throughout Thailand.

The public LTC system benefits different services in different countries, in terms of the socio-economics, source of funding, care administrators, tax structure, or even social capital. The management of LTC in developed countries (for example, Germany, the United States, Japan, the Netherlands) is financed through a combination of government support and copayments from beneficiaries, while the system in Thailand is fully funded through public financing schemes.

In Thailand, LTC may be considered a suitable service for the elderly for two main reasons. The first reason is that it is cost-effective compared to hospital-based care, especially for chronic or palliative care [9] Chandoevwit & Vajragupta (2017). The second is that the social capital of several rural communities is capable of supporting the care. With the LTC budget constraints, LTC implementation is always based on collaboration between communities and the government. The Village Health Volunteers and the Elderly Home Care Volunteers will work closely together with the Sub-District Health Promoting Hospitals or local governments to provide essential medical and social support to the elderly with disabilities in their communities.

For example, the role of formal care providers such as sub-district health promotion hospital is to provide functionality assessments for the elderly, create an individual care plan, and submit for the local government's financial support. Before the proactive plan, the Village Health Volunteers already deployed medical staff members in their village to assist with tasks such as health surveys, disease control and prevention, and door-to-door visit. However, in 2016 the government gave additional financial incentives to those volunteers who are willing to take Elderly Rehabilitation training for at least 70 h, providing them with a new role as a paid caregiver. Each caregiver will take care of the elderly people in their community and close monitoring by the specialized.

## 3 Data

The data in the paper is provided from the project of “An Effectiveness Analysis of the Long-Term Care Plans in Udonthani Province” conducted by the Thailand Development Research Institute (TDRI) and supported by the Thai Health Promotion Foundation. The dataset is an annual balance panel from 2016 to 2017, consisting of a total of 279 elderlies over 60 years of age and has ADL scores below 11 (in 2016), which defines as a homebound and bedridden older person. A clustered multi-stage design is used to select the sample from 32 sub-districts (in 10 districts of Udonthani province, Thailand).

Life satisfaction is measured by asking how satisfied people are with their life at present. Responses report scores to this question on a scale of 1–10, in which 1 is 'totally dissatisfied' and ten means “totally satisfied.” Moreover, life satisfaction refers to the consideration of life as a whole, rather than emotions in recent experience. This is consistent with Clark et al. and Helliwell & Putman who state the benefits of using life satisfaction as a single variable to represent subjective wellbeing as a whole are that life satisfaction is more stable reflects long-term life change and is broader in scope [10], [11].

The consumer/care-receipt satisfaction in LTC is marked as the proxy variable representing LTC service quality. The satisfaction in LTC is considered by asking a question: “How satisfied are you with LTC service?”. The scores are ranked from 1 to 10 and can be interpreted as follows: 1 is “totally dissatisfied”, and ten means “totally satisfied”. [12] Dubina and Feldman found that the physician’s interpersonal skills, concern for the patient's health correlate with a hider rate of care-receipt satisfaction. Therefore, frequent follow-up visits, spending time with the patient, and give an explanation of treatment are viable ways to raise care-receipt satisfaction.

The independent variables in our life satisfaction model are categorized into four components: 1) socioeconomic variables, 2) physical health, 3) social interaction, and 4) social support variable [11], [13], [14]. LTC service represents component 4, and the other control variables include gender, age, schooling, household income, stay together with the spouse, chronic diseases, social participation, and ADL scores. The ADL index is the variable used to measure the health conditions, representing how well the elderly can perform basic self-care tasks such as dressing, bathing, climbing stairs, mobility, feeding, and toilet use [15], [16] Shelkey and Wallace suggest that the ADL index is the appropriate instrument to assess functional status, but they may have a limitation in measuring small increments of change in physical conditions. Table 1 provides details on the variables that we use for our econometric analysis, also the mean value and standard deviation are presented for each variable.

**Table 1:** Data and Statistics Descriptive.

Variable	Description	Mean	Min	Max	S.D.
Life Satisfaction	How satisfied are you with your life at present? On scale 1–10	6.746	1	10	2.323
Care-receipt Satisfaction in LTC	How satisfied are you with LTC service? On scale 1–10	7.897	1	10	2.098
Male	Dummy variable equals 1 if respondent is male, 0 if respondent is female.	0.356	0	1	0.479
Age	Age (ratio scale)	78.089	60	104	9.172
Schooling	Dummy variable equals 1 if respondent attends school, 0 if respondent doesn't attend school.	0.168	0	1	0.374
Household Income	Log of household income	11.448	9	14	0.821
Stay with Spouse	Dummy variable equals 1 if respondent lives together with a spouse in the same house, 0 if respondent and spouse don't live together or the spouse is deceased.	0.405	0	1	0.491
Chronic-diseases	Dummy variable equals 1 if respondent has chronic diseases, 0 if respondent doesn't have chronic diseases.	0.702	0	1	0.457
ADL	The Barthel Index of Activities of Daily Living (ADLs) (scale 0–11 in the year 2016)	6.577	0	20	4.637
Social Participation	Dummy variable equals 1 if respondent participates in any social events (such as religion and community activity) within one year, 0 if respondent doesn't participate.	0.112	0	1	0.316
Home Visit	Dummy variable equals 1 if home visit is provided by government staff within a year, 0 if respondent doesn't receive any home visit from government staff.	0.870	0	1	0.335
Care Time	Length of time for visits in the last week (minutes/week)	24.856	0	840	76.762
Proactive LTC	Dummy variable equals 1 if respondent receives proactive LTC service, 0 if respondent receives only the public standard LTC.	0.358	0	1	0.479

Note: data sample consists of a two-year panel of 279 individuals.

Other determinants of happiness of elderly people are mentioned in the studies below [17]. Gwozdz and Sousa-Poza conducted a study of the elderly aged 75 years and over in Germany and found that health condition is a crucial variable of life satisfaction. There is evidence confirming that social interaction is closely related to elderly happiness ([18], [19] and health condition [20], [21], [23]. In terms of income effect, [20] Clark et al. find that household income can create only a small part of the impact on life satisfaction. It is worth noticing that the homebound and bedridden elderly face difficulty with their daily routines and need more support than the active elderly, so the key to the happiness of those may be different from the findings of previous studies.

Table 2 displays the distribution of health conditions and LTC proceeding in Udonthani for 2016 and 2017. Compared to the year 2016 and 2017, there is not much change in the number of bedridden, the year 2016, there is 36.6% of the respondents are bedridden, while in the year 2017, the homebound seem to be healthier (24.4% become active elderly). [22], [24] mention that changes in frailty transition patterns may be associated with age and follow-up periods; however, since our data is a 2-year panel, it shows just a small change in the transition. The plurality of the respondents (25.8–28%) rate their satisfaction with LTC giving a score of 10, which means “totally satisfied.” The number of elderly receiving home visits increases from 81% to 93% in the consecutive year since the government generates more funding to expand access to LTC. Most of the Thai people are covered under one of three health care schemes: (1) the civil service welfare system for government employees, (2) the social security scheme, and (3) Universal Health Coverage Scheme. Table 2 shows that most of the respondents are under the third scheme. The average length of time for home visits by government staff in the last week is about 25 min per visit. We found that the higher the average length of time for home visits, the higher score of satisfaction. For example, at an average of 37 min per home visit, people score LTC (9–10) satisfaction, while at an average of 11 min home visits, people score LTC (1–5) satisfaction.

**Table 2:** Distribution of health condition and LTC related variables for 2016 and 2017.

	2016		2017	
	N	%	N	%
**Health condition**				
ADL 0–4 (Bedridden)	102	36.6	103	36.9
ADL 5–11 (Homebound)	177	63.4	108	38.7
ADL more than 12 (only in 2017)			68	24.4
**Long term care policy**				
Proactive LTC has not implemented	179	64.2	179	64.2
Proactive LTC has implemented	100	35.8	100	35.8
**Care-receipt Satisfaction in LTC**				
Below 4	55	20.0	71	25
5	27	9.7	16	5.7
6	20	7.2	17	6.1
7	34	12.2	25	9.0
8	50	17.9	40	14.3
9	21	7.5	32	11.5
10	72	25.8	78	28.0
**Home visits**				
Not receive any home visit within a year	53	19.0	19	6.8
Receive at least one home visit within a year	226	81	260	93
**Type of health insurance plan**				
Government employee	26	9	24	9
Social security	1	0	0	0
Universal Health Coverage Scheme,	252	90	253	91
Not used at all			1	0

**Fig. 1:**
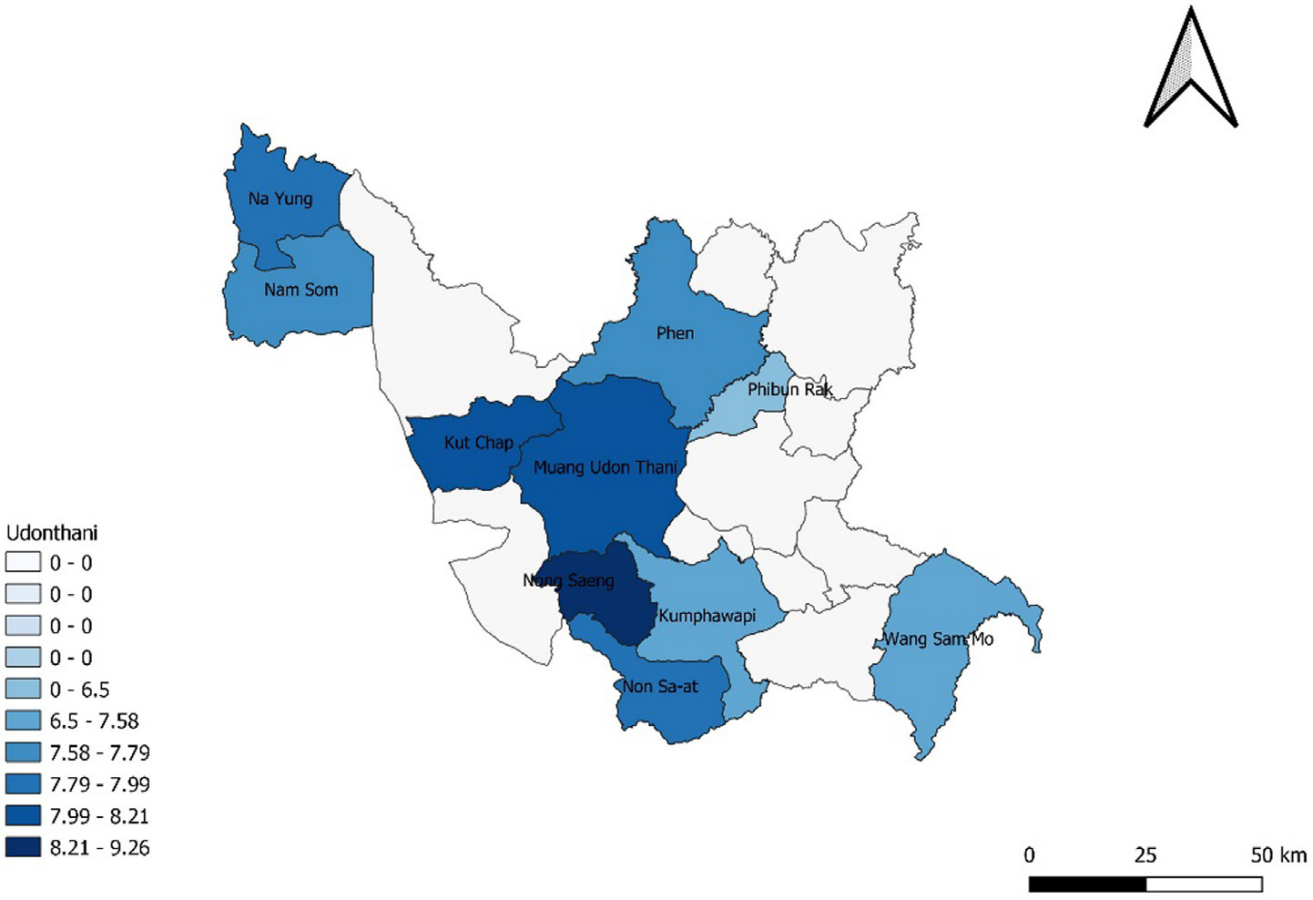
Care-receipt satisfaction in LTC for homebound and bedridden elderly.

**Fig. 2:**
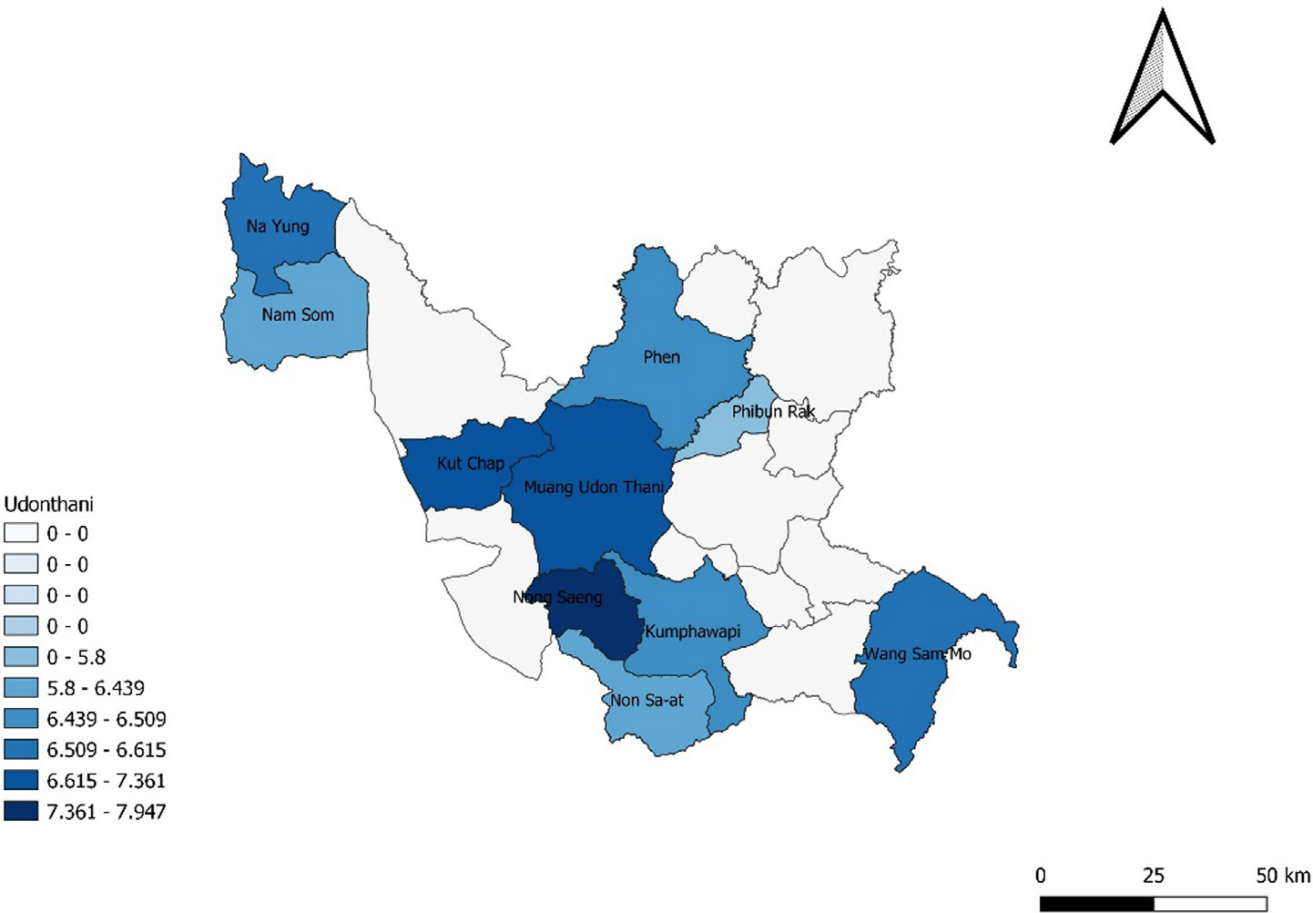
Average life satisfaction for homebound and bedridden elderly.

Table 3 presents the mean of life satisfaction by care-receipt satisfaction in LTC. The data brings preliminary support to the idea that lower satisfaction in LTC is generally associated with lower life satisfaction. Fig. 1, Fig. 2 present the data from table 3 in a more visual manner. The darker blue districts represent areas with higher LTC satisfaction and life satisfaction, respectively. In the next section, we will also take into account other relevant factors that are likely to influence life satisfaction.

**Table 3:** Mean life satisfaction by care-receipt satisfaction in LTC.

Care-receipt Satisfaction LTC (score 1–10)	Average Life Satisfaction	no	%
10	9.25	76	13.62
9	9.00	45	8.06
8	8.21	70	12.54
7	7.79	53	9.49
6	7.42	57	10.22
5	7.24	94	16.85
4	6.67	21	3.76
3	7.05	22	3.94
2	6.67	12	2.15
1	5.25	8	14.33
Reject or not be able to answer		100	17.92

## 4 Empirical methods

We treat the life satisfaction variable as a continuous outcome. As we described earlier, this paper uses an annual balance panel from 2016 to 2017, consisting of a total of 279 dependent elderly people over 60 years of age, which has both cross-sectional and time-series dimensions. It can be said that the panel data provides more flexibility in modeling differences in behavior across individuals over time [25]. The estimation techniques for panel data with continuous dependent variables commonly use fixed and/or random-effects models. The former allows for a correlation between the individual effect and the regressors of the model, while the latter assumes the unobserved factors are uncorrelated with the regressors and the overall disturbance term [26].

Due to endogeneity issues, the follow-up visits and time spending of medical staff might correlate with care-receipt satisfaction, support the idea of treating satisfaction in LTC as an endogenous variable [12], [27] The endogeneity issues arise due to a non-zero correlation between individual unobserved effects and explanatory variables [28]. However, the endogeneity biases cannot be removed via differencing or fixed effects estimation, and hence, require special consideration [29], [30], [31].

Therefore, instead of using only one stage of analyzing the impact of LTC satisfaction by fixed or random-effects models, this paper conducts an analysis of the pooled two-stage least squares (pooled 2SLS) and fixed effects-2SLS (FE-2SLS) estimators and discuss complications. We create the life satisfaction model from Suriyanrattakorn and Awaworyi et al. [32], [33], which are as the following:

$${\mathrm{LS}}_{\mathrm{it}}={\beta LTC}_{\mathrm{it}}+\gamma X_{1it}+\mu_{i}+v_{\mathrm{it}}$$

$${\mathrm{LTC}}_{\mathrm{it}}=Z_{\mathrm{it}}\delta +\mu_{i}+v_{\mathrm{it}}$$

where, i=1,2,⋯,n, t=1,2, $v_{i}$is a random error term that is independent and identically distributed over the panels. $X_{1it}$ is a set of control variables including gender, age, schooling, household income, cohabitation with a spouse, chronic-diseases, ADL scores, and social participation. $\mu_{i}$ represents the unobservable individual heterogeneity.

${\mathrm{LS}}_{\mathrm{it}}$ is the life satisfaction for individual i in periodt. ${\mathrm{LTC}}_{\mathrm{it}}$ is the measure of care-receipt satisfaction in LTC, which is treated as an endogenous variable and is allowed to be correlated with the error term $v_{\mathrm{it}}$. In this case, we instrument the LTC variable using three variables; (1) home visit provided by government staff, (2) receiving a proactive LTC service, and (3) length of time for the home visit. We treat gender, age, schooling, household income, stay together with the spouse, chronic-diseases, ADL scores, and social participation as exogenous variables.

## 5 Empirical results

Table 4 illustrates the impact of care-receipt satisfaction in LTC on overall life satisfaction from five different estimation methods. Columns 1, 2, and 3 present pooled ordinary least square (OLS), fixed effect (FE), and random effect (RE) results. Columns 4 and 5 present pooled two-stage least square (Pooled-2SLS) and random effect two-stage least square (RE-2SLS) estimates. In models (4) and (5), we treated the satisfaction in LTC as an endogenous variable, and the instrument for care receipt satisfaction with LTC is determined by the home visits, receiving a proactive LTC service, and length of time for the home visit. The bottom of Table 4 shows the result of the first stage relationship. We find a positive relationship between home visits, length of time for the home visit, and LTC satisfaction.

**Table 4:** Care-receipt satisfaction in LTC and life satisfaction.

	(1)	(2)	(3)	(4)	(5)
Variables	Pooled OLS	FE	RE	Pooled-2SLS	RE-2SLS
Care-receipt Satisfaction in LTC	0.460***	0.345***	0.456***	0.313*	0.320*
	(0.051)	(0.074)	(0.051)	(0.177)	(0.177)
Male	−0.010	1.029**	−0.012	−0.027	−0.027
	(0.239)	(0.419)	(0.250)	(0.237)	(0.250)
Age	0.005	0.212	0.005	0.004	0.004
	(0.012)	(0.216)	(0.012)	(0.012)	(0.013)
Schooling	0.209	−0.593	0.208	0.157	0.159
	(0.257)	(0.593)	(0.232)	(0.256)	(0.241)
Log Household Income	0.325***	−0.009	0.334***	0.339***	0.331***
	(0.120)	(0.256)	(0.119)	(0.120)	(0.120)
Stay with Spouse	0.132	1.632**	0.146	0.182	0.192
	(0.245)	(0.683)	(0.255)	(0.244)	(0.255)
Chronic-diseases	−0.314	−0.629	−0.309	−0.308	−0.304
	(0.228)	(0.713)	(0.235)	(0.227)	(0.237)
ADL	0.040**	0.035	0.040*	0.045**	0.045*
	(0.021)	(0.049)	(0.022)	(0.022)	(0.024)
Social Participation	0.234	0.149	0.237	0.263	0.260
	(0.296)	(0.426)	(0.271)	(0.301)	(0.279)
Constant	−1.327	−13.172	−1.244	−0.111	−0.131
	(1.555)	(16.164)	(1.482)	(2.065)	(2.009)
Observation	458	458	458	458	458
R-squared	0.203	0.039	0.203	0.186	0.198
Hausman Test			10.54		
			[0.309]		
First stage results for Care-receipt Satisfaction in LTC					
Home Visit				1.194	
				(0.278)***	
Care Time				0.005	
				(0.001)***	
Proactive LTC				0.002	
				(0.199)	
Durbin Chi-square Test				0.836	
				[0.361]	
Wu-Hausman F Test				0.767	
				[0.382]	
Stock-Yogo F-statistics				10.596	
				[0.000]	
Sargan Test (chi-square)				2.246	

Note: White's heteroskedasticity-robust standard errors are given in parentheses, *, **, and ** denote significance at 10%, 5%, and 1%, respectively. The value in [–] represents p-value.

Before interpreting the causality of the explanatory variables to life satisfaction, we conduct multiple statistical testing (such as the Hausman test and endogeneity tests) to check which models (Pooled OLS, FE, RE, pooled-2SLS, and RE-2SLS) are appropriate for this data. Firstly, we use the Hausman test to decide if we should use FE or RE. Under the null hypothesis of no correlation between regressors and effects, the Hausman test cannot reject the null hypothesis, which indicates that RE is the preferred model.

Next, we perform the tests for endogeneity, the relevance of the exogenous variable, and over-identifying restrictions of two-stage least square regression. The result of the [34] Stock-Yogo test shows that the critical value of the first-stage F-statistic is 10.596, so the null hypothesis (the instruments are weak) is rejected, which implies the LTC instrument variables are relevant enough to be included. Sargan test is used to test the identifying restrictions for the instrument variables that are uncorrelated with the error term. The results show an insignificant chi-square statistic value (2.246), which implies our model is rather accurate. However, when we further use the Durbin and Wu–Hausman tests to detect the endogeneity of LTC satisfaction, the statistics cannot reject the null hypothesis that LTC is exogeneity, which also indicated that instrumental variable estimation would be less efficient.

Therefore, according to the test results above, the RE model is preferable to the RE-2SLS model. However, we find that the results among the five models are considerably consistent with each other in terms of significance and sign of the coefficient. The vital finding proofs that satisfaction in LTC generates a significantly positive impact on overall life satisfaction of the homebound and bedridden elderly at the 0.01 significant level for RE and pooled OLS, as well as pooled-2SLS and RE-2SLS estimations. This means that a unit increase in care receipt satisfaction level causes an approximately 0.456–0.460 units increase in overall life satisfaction. Besides, we discover that homebound and bedridden elderly's life satisfaction also depends on the health and financial conditions, which means the higher income or ability to perform self-care, the greater the possibility of being satisfied with their life. Our result is consistent with Gwozdz and Sousa-Poza [17], who points out that health condition is the significant key variable affecting senior life satisfaction.

Considering the effect of personal background on life satisfaction, the results show no significant differences in gender, age, schooling, and chronic-diseases to a rating of one's life satisfaction level. Also, social participation does not indicate any significant relationship with life satisfaction in all the estimation methods. Even though previous studies [11], [20] discovered that social participation can positively impact life satisfaction. This can be explained by the difficulty with the mobility of the dependent elderly. The data shows that only 3% of the respondents are willing and can participate in social events. This might cause an insignificant relationship between social participation and life satisfaction.

## 6 Conclusion and discussions

The number of homebound and bedridden elderly has been increasing in Thailand as the aging population rapidly grows and rates of chronic diseases increase. As a result, Thailand requires LTC development to make dependent elderly more satisfies with the care and hopefully contribute a positive effect on their overall happiness. Using a two-year panel of 279 dependent elderlies, we find that homebound and bedridden elderly's happiness can be explained with the casual effect both from public policy and their factors. We find that a higher degree of LTC satisfaction, which describes better quality LTC, can deliver a positive effect for the homebound and bedridden elderly in terms of enhancing their overall happiness. We also discover that the elderly's happiness depends on financial status and how well they perform in their activities of daily living.

The importance of LTC policy is stated in Sustainable Development Goals (SDGs), Goal 3 as a tool to ensure healthy lives and promote wellbeing for all at all ages. Our empirical result supports the vital role of LTC service in order to improve the subjective wellbeing of the elderly. The policy recommendations derived from these results can suggest that the Thai proactive LTC approach may be on the right track to extend LTC service accessibility to cover more target groups across Thailand. However, the quality of the service should be a significant concern to improve the happiness of the dependent elderly. The key takeaways from this study, based on the case study in Udontani, can highlight the importance of finding tools that can improve LTC satisfaction to improve dependent elderly's happiness in Thailand

It is worth noticing that although the first stage of our regression shows the impact of home visits and length of time for home visits on LTC satisfaction. The endogeneity test has shown that an instrumental variable estimation is less efficient compared to the based models. Therefore, we cannot be certain that an increase in the frequency of home visits or time spent will be an adequate method to improve LTC satisfaction. Further research must be conducted to confirm the causal effect of explanatory variables towards LTC satisfaction.

## CRediT authorship contribution statement

**Savinee Suriyanrattakorn:** Conceptualization, Data curation, Software, Writing - original draft. **Chia-Lin Chang:** Supervision, Methodology, Software.

## Declaration of Competing Interest

The authors declare that they have no known competing financial interests or personal relationships that could have appeared to influence the work reported in this paper.

## References

[1] Foundation of Thai Gerontology Research and Development (TGRI). Situation of Thai Elderly; 2016 [cited 2019 Dec 5]. Available from: http://www.dop.go.th/download/knowledge/th1512367202-108_0.pdf.

[2] Prakash B. (2010). Patient satisfaction. J Cutaneous Aesthetic Surg.

[3] Matsuguma S., Negishi K., Kawashima M., Toda I., Ayaki M., Tsubota K. (2018). Patients’ satisfaction and subjective happiness after refractive surgery for myopia. Patient Prefer Adherence.

[4] Roberts R.E., Pascoe G.C., Attkisson C.C. (1983). Relationship of service satisfaction to life satisfaction and perceived well-being. Evaluat Prog Plann.

[5] Kane R.L., Kane R.A. (2001). What older people want from long-term care, and how they can get it. Health Aff.

[6] Dolan P., Metcalfe R. (2012). Measuring subjective wellbeing: recommendations on measures for use by national governments. J Soc Policy.

[7] Abruquah L.A., Yin X., Ding Y. (2019). Old age support in urban China: the role of pension schemes, self-support ability and intergenerational assistance. Int J Environ Res Public Health.

[8] Sasat S., Choowattanapakorn T., Pukdeeprom T., Lertrat P., Aroonsang P. (2014). Long-term care institutions in Thailand. J Health Res.

[9] Chandoevwit W., Vajragupta Y. (2017).

[10] Clark A.E. (2018). Four decades of the economics of happiness: where next?. Revier Income Wealth.

[11] Helliwell J.F., Putnam R.D. (2004). The social context of well–being. Philos Trans Roy Soc B: Biol Sci.

[12] Dubina M.I., O'Neill J.L., Feldman S.R. (2009). Effect of patient satisfaction on outcomes of care. Exp Rev Pharmacoecon Outcomes Res.

[13] Blanchflower D.G., Oswald A.J. (2004). Well-being over time in Britain and the USA. J Public Econ.

[14] Easterlin R.A., McVey L.A., Switek M., Sawangfa O., Zweig J.S. (2010). The happiness–income paradox revisited. Proc Natl Acad Sci.

[15] Mahoney F.I., Barthel D.W. (1965). Functional evaluation: The Barthel Index: a simple index of independence useful in scoring improvement in the rehabilitation of the chronically ill. Md State Med J.

[16] Shelkey M., Wallace M. (2000). Katz Index of Independence in Activities of Daily Living (ADL). Dir Cincinnati Ohio.

[17] Gwozdz W., Sousa-Poza A. (2010). Ageing, Health and Life Satisfaction of the Oldest Old: An Analysis for Germany. Soc Indic Res Int Interdiscip J Qual-Life Meas.

[18] Nanthamongkolchai S., Tuntichaivanit C., Munsawaengsub C. (2009). Factors influencing life happiness among elderly female in Rayong Province, Thailand. J Med Assoc Thail Chotmaihet Thangphaet.

[19] Kramanon R., Gray R.S. (2015). Differentials in happiness among the young old, the middle old and the very old in Thailand. J Popul Soc Stud.

[20] Clark A.E., Frijters P., Shields M.A. (2008). Relative Income, Happiness, and Utility: An Explanation for the Easterlin Paradox and Other Puzzles. J Econ Lit.

[21] Helliwell J.F., Layard R., Sachs J. (2019).

[22] Clark A.E., Flèche S., Layard R., Powdthavee N., Ward G. (2018).

[23] Headey B., Wooden M. (2004). The effects of wealth and income on subjective well-being and ill-being. Econ Rec..

[24] Kojima G., Taniguchi Y., Iliffe S., Jivraj S., Walters K. (2019). Transitions between frailty states among community-dwelling older people: a systematic review and meta-analysis. Ageing Res Rev.

[25] Greene W.H. (2002).

[26] Chamberlain G. (1980). Analysis of covariance with qualitative data. Rev Econ Stud.

[27] Gu N.Y., Gai Y., Hay J.W. (2008). The effect of patient satisfaction with pharmacist consultation on medication adherence: an instrumental variable approach. Pharmacy Practice.

[28] Wooldridge J.M. (1995). Selection corrections for panel data models under conditional mean independence assumptions. J Economet.

[29] Semykina A., Wooldridge J. (2010). Estimating panel data models in the presence of endogeneity and selection. J Appl Economet.

[30] Kyriazidou E. (1997). Estimation of a panel data sample selection model. Econometrica.

[31] Rochina-Barrachina M.E. (1999). A new estimator for panel data sample selection models. Annales d'Economie et de Statistique.

[32] Suriyanrattakorn S. (2019). Happiness among the disabled elderly: a study based on micro data in Udonthani Thailand. Thammasat Econ J.

[33] Awaworyi Churchill S., Smyth R. (2019). Transport poverty and subjective wellbeing. Transp Res Part A Policy Pract..

[34] Stock J.H., Yogo M., Andrews Donald W.K., Stock James H. (2005). Identification and Inference for Econometric Models: Essays in Honor of Thomas Rothenberg.

